# Trust in the Leader, Organizational Commitment, and Nurses’ Intention to Leave—Insights from a Nationwide Study Using Structural Equation Modeling

**DOI:** 10.3390/nursrep14020109

**Published:** 2024-06-10

**Authors:** Dhurata Ivziku, Valentina Biagioli, Rosario Caruso, Marzia Lommi, Anna De Benedictis, Raffaella Gualandi, Daniela Tartaglini

**Affiliations:** 1 Department of Health Professions, Fondazione Policlinico Universitario Campus Bio-Medico, 00128 Rome, Italy; d.tartaglini@policlinicocampus.it; 2 Department of Medical and Surgical Sciences—DIMEC, University of Bologna, 40138 Bologna, Italy; valentina.biagioli@unibo.it; 3 Health Professions Research and Development Unit, IRCCS San Donato Hospital, San Donato Milanese, 20097 Milano, Italy; rosario.caruso@unimi.it; 4 Department of Biomedical Sciences for Health, University of Milan, 20133 Milano, Italy; 5 Department of Biomedicine and Prevention, Tor Vergata University, 00131 Rome, Italy; marzia.lommi@alumni.uniroma2.eu; 6 Clinical Directory, Fondazione Policlinico Universitario Campus Bio-Medico, 00128 Rome, Italy; a.debenedictis@policlinicocampus.it; 7 Research Unit in Nursing Science, Universitá Campus Bio-Medico di Roma, 00128 Rome, Italy

**Keywords:** nurses, intention to leave, leadership, organizational commitment, mediation, structural equation modeling, trust in the leader

## Abstract

Nursing retention is a major challenge globally. Ongoing workforce instability across countries underscores the need to understand the factors influencing turnover and nursing retention. Trust is a crucial element in managing workplace relationships between nurse managers and nurses. Existing studies have shown the direct impact of trust on employees’ intention to leave their job but have not explored the effects of potential mediators such as organizational commitment. The aim of this study was to examine the impact of trust in the leader on nurses’ intention to leave their job through the mediation of organizational commitment. A cross-sectional study was conducted in Italy. A convenience sample of 1853 nurses completed a self-report survey. The study tested a hypothesis-based mediation model using structural equation modeling, which showed good fit indices. The results indicated that trust in the leader had a significant impact on nurses’ intention to leave, and this relationship was partially mediated by organizational commitment. Nurses who trust their leader are more likely to demonstrate higher levels of organizational commitment, resulting in a lower intention to leave their job. Furthermore, organizational commitment and trust emerge as critical factors in reducing nurses’ intention to leave their current positions. Therefore, managers can reduce nurses’ intention to leave by building trustful relationships that enhance organizational commitment.This study was not registered.

## 1. Introduction

Healthcare organizations are complex systems marked by a multitude of interconnected elements and stakeholders. They perpetually evolve and adapt to shifting health demands, technological advancements, and resource dynamics. In navigating this complexity, managerial leadership has a critical role in driving organizational success and facilitating adaptability to dynamic healthcare landscapes. 

Managers indeed play a crucial role in guiding nursing performance, ensuring the delivery of quality care to achieve better patient outcomes [1]. In fact, a recent meta-review documented that nurse managers’ relational leadership styles were associated with enhanced patient satisfaction and quality of care, an improved safety climate, reduced errors, falls, pressure ulcers, and adverse events [2]. Similarly, positive organizational outcomes, including outcomes related to organizational culture, trust, citizenship behavior, supportive working conditions, and safe practices, were observed when nurse managers consistently employed relational leadership styles [2]. Conversely, poor management practices can lead to workplace incivility and negative outcomes associated with nursing performance [1]. 

The quality of managerial leadership has a significant impact on the well-being of healthcare teams and their job-related outcomes. Moreover, high-quality leadership fosters positive work environments, which are associated with a 28–32% reduction in the odds of job dissatisfaction, burnout, or intention to leave [3], facilitating improvements in organizational commitment and, consequently, reductions in absenteeism and turnover intentions [4,5]. Alternatively, nurses exposed to a nurse manager with toxic behaviors reported increased intentions to leave their organization and profession [1,2,6].

Collaborative and supportive interactions form the foundation of relational leadership styles. Trust is a key factor in fostering positive workplace relationships. The interest in exploring trust in healthcare has been limited, but it has surged notably in the last two decades [7]. From a work environment perspective, trust has been examined from two angles: trust in the leader and trust in the organization [7]. Within the healthcare literature, trust has been identified as a mediator between authentic [8], empowering [9], ethical [10], and transformational [5] leadership styles and employees’ psychological states, well-being, performance, commitment, satisfaction, and intention to leave [2]. In addition to such mediation effects, other studies conducted among nurses have tested the direct effects of trust on various outcomes, including organizational commitment [7,11,12], job satisfaction [7,11,13], work–life balance [13], and well-being [7,14,15]. Furthermore, trust in the leader has been associated with productivity [14], collaboration and communication [7,11], and work engagement [16]. In addition, trust emerges as a determinant affecting nurse turnover intentions [17]. Understanding how trust dynamics in healthcare settings affect social interactions is essential for cultivating positive work environments that promote cooperation, collaboration, and productivity [18] and for retaining nurses [2].

Although trust has been extensively studied across diverse disciplines, there is no universal definition of trust [7,14]. Trust is a multidimensional construct characterized by the follower’s positive belief or perception of the relationship with the leader [7], which dynamically fluctuates in intensity and significance over time, and is intricately linked to the context [19]. The facets of trust include a range of cognitive, emotional, and behavioral aspects, such as honesty, reliability, confidence, respect, reciprocity, vulnerability, communication, understanding and expectation, and the quality of interactions [7,19]. Based on Tzafrir et al., [20], this research considered trust according to the following definition: “trust is a willingness to increase one’s resource investment in another party, based on positive expectation, resulting from past positive mutual interactions”. 

Organizational commitment, in addition to trust, exhibits a strong effect on nurses’ turnover intentions [21]. It is an indicator of a harmonious relationship between nurses, nurse managers, and the team. Indeed, commitment enhances intrinsic motivation, which is manifested in nursing performance and the hospital’s effectiveness in achieving organizational objectives [22,23]. Empirical findings demonstrate that nurses’ organizational commitment is influenced by their trust in the healthcare organization, management, and coworkers [24] and mitigates the recruitment–turnover sequences among nursing staff [22]. 

Addressing nurse turnover is crucial to combating the global shortage of nurses, which significantly impacts healthcare settings. A recent study conducted in Italy revealed that 35.5% of nurses expressed the intention to leave their current position, out of which 33.1% indicated their intention to exit the nursing profession altogether [25]. These findings are consistent with those from other countries, where the rate of nurse turnover has been estimated to range from 4% to 54% [26,27]. Both the World Health Organization [28,29] and the International Council of Nurses [30] have suggested a global workforce plan to ensure the long-term viability of the nursing profession. They have recommended urgent action to increase nurse retention rates, optimize deployment, improve the quality of the work environment and workloads, and maximize the utilization of nursing skills. These measures are crucial for enhancing nurse well-being, improving patient safety, and enhancing the quality of nursing care. 

Nurses’ intention to leave is a strong predictor of turnover [26]. Younger nurses appear to be those more willing to leave their jobs [25]. Age and generational differences among nurses concerning values, communication styles, and workplace expectations have been reported [31]. The disparity between the expectations and actual experiences of newly licensed nurses has been identified as a transition shock, contributing to increased turnover rates [31]. A meta-analysis, drawing from 106 primary studies, found that supportive leadership, organizational commitment, and network centrality were the most significant predictors of turnover [32]. Another systematic review of the literature revealed that stress, burnout, and job dissatisfaction were key determinants of turnover at the individual level, with supervisor support being crucial for retaining employees [26]. The authors suggested that understanding the work environment and dynamic interplay between variables could help address the issue of nurses intending to leave their jobs [26,32]. It has been recommended that further research be carried out to discern the interactive and mediating effects of variables associated with nurses’ intention to leave [26]. Hence, understanding the factors that influence nurses’ intention to leave, including their interactions and mediating effects, is paramount.

Therefore, this study aims to contribute to the existing literature on nursing management by examining the impact of trust in the leader on nurses’ intention to leave their jobs through the mediation of organizational commitment. To the best of our knowledge, no prior studies in the nursing literature have examined the proposed direct and indirect associations. We used a hypothesis-based model to improve our understanding of the relationship between trust in leadership, organizational commitment, and nurses’ intention to leave. This may help nurse managers and healthcare institutions identify key factors to mitigate turnover rates and enhance patient care outcomes.

**Figure 1:**
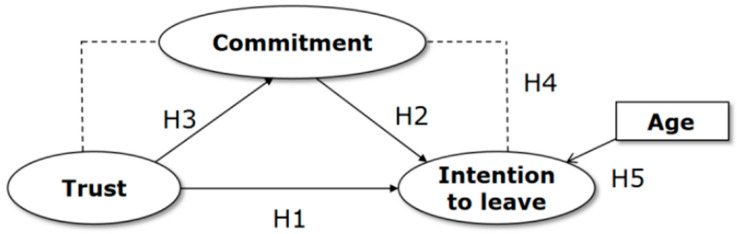
The hypothesis-based research model.

Based on our literature review, we formulated the following hypotheses presented in the model in Figure 1:

**Hypothesis** **1.** *Trust in the leader has a direct negative effect on nurses’ intention to leave.*

**Hypothesis** **2.** *Organizational commitment has a direct negative effect on nurses’ intention to leave.*

**Hypothesis** **3.** *Trust has a direct positive effect on organizational commitment.*

**Hypothesis** **4.** *Organizational commitment mediates the relationship between trust in the leader and nurses’ intention to leave.*

**Hypothesis** **5.** *An increase in age will result in a decrease in nurses’ intention to leave.*

## 2. Materials and Methods

### 2.1. Design

For this study, we used a cross-sectional descriptive design. The research followed the STROBE (Strengthening the Reporting of Observational Studies in Epidemiology) guidelines for observational studies [33].

#### Theoretical Framework

Social Exchange Theory (SET) [18] provides conceptual perspectives valuable to the field of management and its understanding. It elucidates the complex dynamics of workplace relationships and behaviors, as well as the subsequent actions or inactions of followers and their reciprocal relational responses [34,35]. According to SET, positive leadership styles and actions (behavior) will elicit (action/reaction) increased trust (behavior) from followers, which, in turn, prompts positive behavioral responses such as enhanced organizational commitment (response) and reduced turnover intentions (response). Empirical research, as previously described, indicates that trust and the quality of leader–member exchange (LMX) are critical organizational factors that impact followers’ reactions and responses regarding work attitudes and performance outcomes [36]. 

In an effort to contribute to the understanding of the relationships between Italian nurses and nurse manager samples, a research team is exploring various connections between nurse manager leadership and nurses’ outcomes [36,37,38]. In this study, we aimed to investigate whether trust in the leader could be understood as an expected behavior within the framework of social exchange theory, with organizational commitment serving as both a potential response to the leader’s trustful behavior and as an antecedent of nurses’ intention to leave.

### 2.2. Sample and Setting

In this study, the authors used maximum variation sampling, inviting nurses from diverse healthcare organizations across numerous regions in Italy to participate in the study. The aim was to investigate workplace relational dynamics in a nationwide context. Therefore, registered nurses employed by public and private healthcare organizations, working in hospitals, community care centers, and home care services, were invited to participate in the research.

Participation in the research was voluntary and anonymous, and sampling was convenience-based. Registered nurses were included in the research if they (a) were employed in a public or private healthcare organization, (b) had collaborated with other healthcare professionals in a team, and (c) had a minimum of one year work experience in the service. The exclusion criteria encompassed the following: (a) working independently as a registered nurse, (b) working alone not within a team, (c) not being allocated to a consistent work environment, and (d) returning to service for less than two months after an extended absence.

An a priori sample size was calculated using G*Power 3.1 (Heinrich Heine University). A recommended minimum sample size of 1713 participants could achieve 80% power, enabling the use of structural equation models using 22 observed variables and 6 latent variables, with an anticipated effect size of 0.10 [39] and a level of significance *p* < 0.05. Nevertheless, we recruited 1856 participants to ensure a more robust analysis. 

### 2.3. Data Collection

The research was proposed to the Italian Scientific Society for the Direction and Management of Nursing (SIDMI) network. The lead researchers (DI and DT) collaborated with interested Directors of Nursing, who then designated local representatives to promote the study among registered nurses at each center, encouraging their participation. Local representatives fostered participation through repeated rounds of study promotion with nurse managers and nurses, along with frequent reminders. An online survey was proposed for data collection, and the link was sent to registered nurses via institutional email addresses. Data collection occurred between August 2022 and December 2023. Principal investigators and local contacts continuously exchanged feedback on participation rates, and various reminders were sent throughout the study.

### 2.4. Measurements

The initial section of the survey gathered socio-demographic data, encompassing age (in years), gender (male, female, other), highest level of education attained, years of work experience, employment site, and healthcare organization typology. 

The second section of the survey explored registered nurses’ intentions regarding leaving their current unit of work, healthcare organization, or nursing profession and was assessed through three single-item measures with dichotomous response options: 0 (no, I do not intend to leave the unit/organization/profession) and (yes, I intend to leave the unit/organization/profession within the next six months). The reason behind utilizing single-item measurements to evaluate a specific construct is based on the literature [40].

The third part assessed workplace relational dynamics. Organizational commitment refers to the extent to which nurses identify with and engage in their organization [41]. The Questionnaire on Experience and Work Evaluation (QEEW 2.0© SKB) [42] incorporates the Organizational Commitment Scale, a self-assessment tool comprising six items rated on a 5-point Likert scale, where 0 denotes strongly agree, and 4 denotes strongly disagree. The scale’s scoring ranges from 0 to 100, with lower scores indicating higher levels of organizational commitment. For analysis purposes, the researchers utilized converted scores. The scale demonstrates robust psychometric properties, including an internal consistency of 0.80, and is already accessible in Italian.

To evaluate trust, we employed the Trust Me Scale created by Tzafrir et al. [20]. The scale assesses trust as a multidimensional construct, comprising three factors: harmony, reflecting a sense of belonging and mutual support in manager–employee relationships; reliability, indicating consistency and adherence to established processes in these relationships; and shared concern for others’ well-being, balanced against personal interests. These factors align well with the conceptual frameworks of trust, representing its emotional dimension (harmony), cognitive dimension (shared concern), and behavioral dimension (reliability) [7,19]. The 16 items are self-assessed and rated on a 5-point Likert scale, ranging from 1 (strongly disagree) to 5 (strongly agree). The score is calculated as the mean, with higher scores indicating greater trust. Cronbach’s alpha coefficients suggest a good internal consistency for the scale: 0.85 for harmony, 0.87 for reliability, and 0.80 for concern [20]. The scale was adapted and translated into Italian [37].

### 2.5. Ethical Considerations

The local Ethics Committee granted approval for the research. Subsequently, the Board of Directors of each participating healthcare organization was approached to seek local approval for the research. The study complied with ethical standards and upheld the principles delineated in the Declaration of Helsinki [43]. Prior to their involvement in the study, all participants received detailed information regarding the research objectives, study procedures, and data handling and provided online informed consent. The study emphasized ensuring the privacy and confidentiality of participants at every stage of the research process. Access to the data was limited exclusively to the research team.

### 2.6. Statistical Analysis

To summarize the demographic and sample data, we employed descriptive statistics. Continuous data underwent normality distribution testing and were expressed as the mean and standard deviation (SD). Ordinal and nominal data were described using frequencies and percentages. Relationships among the variables were tested with Pearson’s correlation coefficients. Initial analysis involved assessing linearity, outliers, missing data, outliers, variance homogeneity, and multicollinearity.

As a first step, we performed a confirmatory factor analysis (CFA) to verify that the items were effectively loaded on their intended constructs and that the study constructs were clearly distinguishable [44]. The CFA was implemented using the robust (unweighted) least squares estimator (ULSMV) method, with the intention to leave as a categorical variable [45]. To test the reliability of the scales, Cronbach α internal consistency coefficient was explored to be within the permissible range [46]. In this study, Cronbach’s alpha values were 0.912, 0.945, and 0.715 for organizational commitment, trust, and intention to leave, respectively.

As a second step, structural equation modeling (SEM) was used to test the hypotheses. The selection of the independent, dependent, and mediation variables was theoretically driven. We specified, in the model, the latent constructs for organization commitment and intention to leave and a second-order latent construct for trust in the leader. We specified trust in the leader as the independent variable, organization commitment as the mediator variable, and Intention to Leave as the dependent variable. To test the mediation hypotheses, we employed specific indirect effects tests using the “Model Indirect” procedure in Mplus 8.4.

The goodness of fit values were assessed in accordance with recommendations from the literature [47,48,49]. In the CFA and SEM models, the following fit indices were evaluated: χ^2^, root mean square error of approximation (RMSEA), standardized root mean square residual (SRMR), comparative fit index (CFI), and Tucker–Lewis index (TLI). Thresholds indicating acceptable model fit were considered based on established criteria: RMSEA < 0.08 (acceptable) and <0.05 (good fit), SRMR < 0.05 (good fit), CFI > 0.95 (good fit), and TLI > 0.90 (good fit). Additionally, traditional chi-square (χ^2^) statistics were employed for assessing model fit [47,49]. In addition, standardized regression coefficients (β) and coefficient of determination values (R^2^) are presented.

All statistical tests were two-sided, and *p* values < 0.05 were considered significant. Missing data for each variable were explored and addressed using available case analysis. Statistical analyses of the data were performed using SPSS software version 27.0 (IBM Corp., 2019, Armonk, NY, USA) and MPLUS v. 8.4.

## 3. Results

### 3.1. Participants

Out of the 2016 participants who were invited to fill out the survey, 1856 submitted completed responses, resulting in a response rate of 92%. No missing data were observed. Table 1 presents the socio-demographic and occupational profiles of the participants. The sample consisted mainly of females (77.2%). Their mean age was 43.4 years (range: 22–66), and their tenure as a nurse was 18 years (SD = 11.4; range 1–45). The majority of responses were received from nurses working in the center (44.1%) and north of Italy (38.2%) and from nurses working in public healthcare organizations (74.4%). A high number of nurses were working on day/night rotation shifts (63%), and 69% held a Bachelor’s Degree in Nursing as their highest education qualification.

The mean scores for trust in the leader for the entire sample were 3.6 (SD = 0.7; range 1–5) for harmony, 3.8 (SD = 0.8; range 1–5) for reliability, and 3.9 (SD = 0.7; range 1–5) for concern. This means that there was a good level of trust between the leader and the group. The mean score for organizational commitment was 49.4 (SD = 21.3; range 0–100), indicating that nurses had a moderate level of commitment toward their organization. Concerning intention to leave, 26.9% of nurses would change their work unit, 22.9% would change organization, and 17.3% would change their profession.

### 3.2. Correlations

We identified significant correlations between the variables studied and the characteristics of the participants (Table 2). Trust in the leader was significantly associated with age, setting, and day shifts, indicating that older nurses, nurses working in public services, and nurses working day shifts had greater trust in their leaders. Regarding organizational commitment, nurses working on day shifts, in public hospitals, and in the southern region of Italy showed higher commitment. An increase in the nurses’ level of education was correlated with reduced organizational commitment. No significant correlations were found for age and tenure. The statistically significant Pearson correlation coefficients were all low (see Table 2 for the coefficients).

Regarding the intention to leave the unit and/or the organization, we found negative associations with shift, age, and tenure as a nurse. This means that younger nurses, those who had been working for a shorter period of time, and those working in rotational shifts (day and night) were more willing to leave their unit and organization. Additionally, the higher the level of education of the nurses, the higher the intention to leave. The only significant correlation with gender was found in the intention to leave one’s unit, which indicated that female nurses were more prone to leave their unit.

As expected, positive medium correlations were found between all the dimensions of trust in the leader and organizational commitment (harmony: r = 0.42, *p* < 0.001; reliability: r = 0.36, *p* < 0.001; concern: r = 0.30, *p* < 0.001). This suggests that the nurses who trusted their leader the most were also committed to their organization. In contrast, a negative correlation was found between intention to leave and trust in the leader (unit: r = −0.27, *p* < 0.001; organization: r = −0.28, *p* < 0.001; profession: r = −0.14, *p* < 0.001), as well as between intention to leave and organizational commitment (unit: r = −0.30, *p* < 0.001; organization: r = −0.46, *p* < 0.001; profession: r = −0.34, *p* < 0.001). These findings indicate that nurses who had lower levels of trust in the leader and reduced organizational commitment were more likely to consider leaving their jobs.

### 3.3. Measurement Model

The CFA was implemented using the ULSMV estimator method. The intended variables univocally loaded the three factors. The loadings for organizational commitment ranged from 0.68 to 0.90, while the intention to leave had loadings ranging from 0.70 to 0.93. Trust in the leader was specified as a second-order factor, with the three dimensions having loadings ranging from 0.56 to 0.90.

### 3.4. Mediation Model

**Figure 2:**
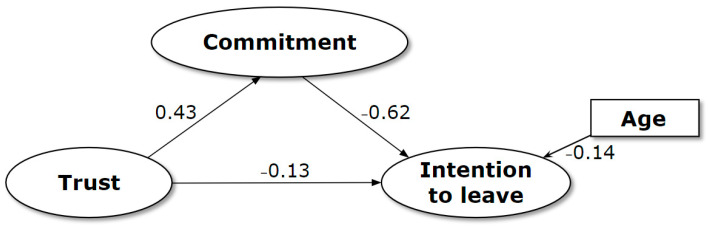
Results of the tested model: the mediation of organization commitment.

The SEM conducted to examine the relationship between trust in the leader, organization commitment, and intention to leave showed satisfactory fit indices: χ^2^ (220; N = 1856) = 1133.20, *p* < 0.001); CFI = 0.93; TLI = 0.92; and RMSEA = 0.05 (90% CI = 0.04–0.05). As shown in Figure 2, trust in the leader positively influenced nurses’ organizational commitment (β = 0.43; *p* < 0.001) and was inversely related to nurses’ intention to leave (β = −0.13; *p* < 0.001). Organizational commitment, in turn, reduced nurses’ intention to leave (β = −0.62; *p* < 0.001). Furthermore, nurses’ older age was associated with reduced intention to leave (β = −0.14; *p* < 0.001). Moreover, organizational commitment partially mediated the relationship between trust in leadership and nurses’ intention to leave (total effect β = −0.39; *p* < 0.001; indirect effect β = −0.26; *p* < 0.001; direct effect β = −0.13; *p* = 0.001). Overall, the model explained 49% of the variance in the intention to leave results. Further details are presented in Table 3.

## 4. Discussion

The main aim of this study was to examine the effects of trust in the leader on nurses’ intention to leave their jobs through the mediation of organizational commitment in healthcare organizations in Italy. Our results confirmed that trust in the leader is a significant determinant of nurses’ intention to leave, and this effect is bolstered by nurses’ organizational commitment. Research on the motivations and influencing factors of nursing turnover intentions in Italy is limited [25,50]. The findings of this study contribute to the national nursing literature and support theoretical development regarding the concepts of trust in organizations and nurses’ turnover intentions [26,27,32]. Specifically, our results extend current research by highlighting the dynamic interplay between trust in the leader and the mediating effects of organizational commitment in mitigating nurses’ intentions to leave their jobs. Therefore, we are broadening the managerial literature on the list of mediators between trust in the leader and intention to leave, which currently only encompasses variables that measure job satisfaction and burnout [21,50], thereby enhancing our comprehension of how nursing work environments affect nurses’ turnover intentions [27,32].

The findings of this study bear substantial relevance for nursing management. Our findings indicate that nurses who place trust in their leaders tend to foster heightened commitment towards their leader, team, and organization, thereby exhibiting a greater propensity to remain within the unit, the organization, and the profession. Our model did not delve into the specifics of managerial leadership style; nevertheless, irrespective of the typology of leadership, nurturing trust within the team emerges as a pivotal factor for enhancing employees’ work engagement [36] and overall well-being [13,18]. Increasing awareness among top and middle managers about the significance of cultivating trusting relationships between managers and nurses to promote a harmonious work environment is vital. This emphasizes the essential role of nurse managers within healthcare organizations and their power, which helps in building quality relationships and stimulating nurses’ organizational commitment and retention. Studies in the literature report that in organizations characterized by a prevailing atmosphere of trust, team members feel safe [36], valued, respected, and supported [11,19], and they also work collaboratively, share knowledge and information [51], improve decision making and performance, reduce time wasting and costs, and increase organizational efficiency [16,19]. Moreover, trustful relationships serve as a potent catalyst for nurse retention, potentially mitigating, to some degree, workload and other environmental factors in fostering stable teams and engaged nurses. Studies in nursing that delve deeper into exploring trust in the relationship between nurse managers and nurses are needed to further expand current knowledge, paying particular attention to nurses in shift rotations who may have fewer opportunities to build relationships with their leaders compared to day-shift nurses.

Managers play a dual role in workplaces as both leaders and supporters of their teams, as well as representatives of the organization [52]. Consequently, a leader’s actions and personal and professional qualities directly influence the commitment and the trust of employees in the workplace but also result in an indirect effect on employees’ organizational trust and commitment. The literature has not yet identified a universally accepted gold standard for effective leadership styles [53]. In healthcare, effective leadership necessitates a manager with a robust leadership education and behavioral attributes capable of fostering strong interpersonal relationships [53] which significantly influence followers’ behavior [37,54,55], including behavior related to their autonomy [38], satisfaction [37,38], organizational commitment, and intention to stay [37], thereby creating better work contexts to motivate the nursing workforce [56,57,58,59,60]. Identified managerial behaviors crucial for fostering trust, as per the conceptual definition of trust [7,19], include active listening, fostering open discussions; encouraging employee involvement [9,60,61], respect [9], empathy and understanding [5,9,61], and personalized support; and acknowledging individuality [5,9,22,60,61]. Additionally, managers should ensure protection against workplace violence and bullying [22], promote engagement and delegate responsibilities effectively [9,16,19,36], express appreciation [22,60], and support professional development [61], particularly by attending to the needs of younger employees [36]. Employees trust managers who are credible, serve as role models, act ethically, are honest [13,60], and foster a supportive organizational culture [2,10,22,36,60]. These leadership behaviors are characteristic of compassionate leadership [60], empowering leadership [9], transformational leadership [5], and servant leadership [36] styles and have been reported to be particularly beneficial during times of organizational stress [22]. While the literature largely describes the beneficial effects of relational leadership styles, nurse managers still encounter difficulties in consistently applying them in their daily practice, especially in crisis or post-crisis situations like the COVID-19 pandemic. Future studies could delve into whether crisis situations could pose a threat to trust relationships between nurse managers and nurses or, conversely, present an opportunity to strengthen or make evident the existence of such trust bonds.

The findings of this study revealed a significant positive association between trust and organizational commitment, thereby adding evidence on these relationships to the limited research on nursing in this area [7]. Previous research on trust has shown that trust is strongly associated with job satisfaction and organizational commitment [24,60]. Indeed, satisfaction and commitment have been recognized as indicators of the harmony between employees and management [62], serving as measures of the strength of employees’ investment in an organization [53]. Organizational commitment enhances efficiency and organization outcomes, reflecting employees’ personal motivation to invest effort in their work performance within the workplace rather than solely focusing on rewards [24,62,63]. Therefore, nurse managers who foster trusting work environments, particularly those who exhibit relational leadership styles [15,23,41], positively influence the organizational commitment of nurses. Additionally, the relationship between trust and commitment has been identified as a factor in reducing turnover among nursing staff, as evidenced by previous research [12,24,63]. This study further validates this phenomenon among Italian nurses, confirming the interactive effects of these two variables on each other [26] and on nurses’ intention to leave.

The topic of nurses’ intention to leave their jobs has received significant attention in the literature, particularly following a notable increase, especially after the COVID-19 pandemic [9,21,22,26]. It has been predominantly examined from a quantitative perspective rather than a qualitative one [64]. In Italy, there are only few studies on nurses’ intention to leave prevalence and the factors related to it [25]. Therefore, exploring the factors that prevent turnover in nursing and the interactions between them is especially crucial in addressing today’s nursing shortage issue [65].

The concept of turnover intention involves a multifaceted and sequential process [26]. Numerous conceptual frameworks which acknowledge a wide range of factors influencing nurses’ turnover or express their intent to do so have been proposed [26]. These factors are typically categorized into three groups: individual, job–work context, and organizational determinants [9,21,26]. However, the specific characteristics of the work context have received comparatively less attention in research [26]. Moreover, turnover risk factors may be influenced by socio-demographic characteristics. In this study, we observed a correlation between increased intention to leave one’s unit and female gender. The literature on turnover and gender contains inconsistent findings that can be attributed to diverse cultural conditions, family structures, and the gender roles of nurses across different settings [22,27]. In our sample, older nurses exhibited higher levels of trust in their leaders and expressed stronger intent to remain in their jobs. Age often correlates with turnover intentions, with less experienced nurses and those with five to ten years of work experience being particularly susceptible. This phenomenon may be explained by the challenges older, more experienced nurses face in changing jobs due to family obligations or a stronger sense of organizational loyalty stemming from a longer tenure [4,22,27,66]. However, other personal factors such as the length of time working in the healthcare environment and in one’s current position did not significantly impact the intention to leave [27]. Our study reinforces the correlation between tenure and job retention, indicating that more experienced nurses tend to stay in their current positions for longer. Finally, the evidence regarding the association between education and turnover intentions remains inconclusive [22,26,27]. In our study sample, we observed that nurses with higher levels of education and those employed in rotating shifts showed an increased intention to leave their jobs. No previous research has explored these aspects; therefore, our research helps to fill this gap. Further research on the effects of socio-demographic factors on nurses’ turnover intentions is warranted.

Reducing nurse turnover [21], enhancing the attractiveness of the profession, and retaining young nurses within the field [25] are essential for healthcare organizations. Within the nursing literature, researchers have explored the determinants of nurses’ intention to leave [21]. However, certain determinants remain underexplored, such as workload, interpersonal conflicts, role identification, job involvement, work self-esteem, team cohesion, and organizational fairness [21,27,30]. Additionally, factors like unfinished or missed nursing care have not received adequate attention in existing reviews [2]. Therefore, future research on nurses’ intention to leave could explore these determinants and other unmeasured mediators.

Effective leaders play a pivotal role in cultivating an environment of trust and commitment, which is essential for retaining skilled nursing staff and enhancing organizational performance [6]. However, the preparedness and suitability of nurse managers for this critical role remain uncertain. The selection process for nurse managers lacks standardization and does not consistently consider their levels of competencies or other relevant indicators [66]. A standardized policy for leader selection in healthcare organizations remains a topic of debate [54,67]. In addition, another significant issue is the departure of nurse managers from their roles. According to the American Organization for Nursing Leadership, challenges with leaders, colleagues, or the organization itself have led many nurse leaders to leave their positions [68]. However, no studies have investigated the relationship between the competencies of nurse managers and their occupational well-being or factors influencing retention [2,69]. Research focusing on these aspects would be valuable, as it could help identify organizational measures to support nursing managers effectively [70].

Ensuring nurse leaders thrive at work is crucial for their ability to positively influence their staff, enhance patient care quality, and organizational performance, as supported by our research. Nurse managers should also focus on promoting the thriving of nurses. Thriving encompasses optimal mental, physical, and social well-being [70]. Despite its importance, the nursing literature lacks comprehensive studies exploring the personal and organizational factors contributing to workplace thriving.

### Strengths and Limitations

This research possesses several notable strengths. Firstly, it included participants from various regions of Italy and diverse healthcare organizations and settings. This breadth of inclusion facilitated greater variability and heterogeneity in the survey data, leading to a more comprehensive depiction of the national situation of nurses in Italy. Additionally, the high response rate and lack of missing data are significant strengths, indicating a strong willingness among nurses to engage in the study and demonstrating that they have a keen interest in the topic. Furthermore, this study marks the first exploration of trust in leadership within the Italian context and is one of the first examinations of mediation effects involving organizational commitment and trust in relation to nurses’ intention to leave.

Nevertheless, it is important to acknowledge the certain limitations of this study. The use of a sample of convenience and the cross-sectional design restrict the generalizability of the findings to all nurses in Italy or beyond. The utilization of a cross-sectional design in this study does not allow for the establishment of causality between the variables to be investigated. Additionally, the reliance on self-report measures introduces the possibility of response biases or social desirability effects. Moreover, we only investigated a subset of variables suggested by the literature that are associated with nurse turnover; hence, we may have overlooked other potential associations. For example, we did not distinguish between turnover rates among nurses in permanent positions and those in temporary roles, nor did we extensively investigate differences among nurses working in public and private healthcare sectors or specific turnover intentions related to the unit, organization, or profession. The primary focus of this research was to explore the effects of the variables included in the study rather than to thoroughly examine the determinants of nurses’ intentions to leave their jobs, as well as aspects of trust and commitment. As discussed, future investigations could delve into leadership styles, the quality of relationships, and other factors to elucidate associations with the variables considered in this research. Consequently, further studies considering a comprehensive range of variables and exploring individual and dyadic (nurse–nurse manager) relationships are warranted to advance our understanding of such factors.

## 5. Conclusions

The findings of this study underscore the critical importance of trust in the leader and organizational commitment in addressing the pressing issue of nursing retention. By examining the mediating role of organizational commitment, our study contributes to unveiling the nexus on nurses’ intention to leave by revealing that trust in leadership positively impacts nurses’ organizational commitment, subsequently reducing their likelihood of considering leaving their current roles. These findings emphasize the need for healthcare organizations to prioritize building trust among their teams and fostering a supportive work environment that encourages organizational commitment. Trustful social interactions are essential for cultivating work environments that promote cooperation, collaboration, and productivity, as well as nurse retention. Ultimately, investing in strategies to enhance leadership and trust-building competencies in nurse managers can yield significant benefits by bolstering nurses’ allegiance to their organizations and reducing turnover intention, thereby promoting organizational stability and improving the quality of patient care.

## Figures and Tables

**Table 1:** Sample characteristics (N = 1856).

	N (%)
**Region**	
North	711 (38.2)
Center	816 (44.1)
South	329 (17.7)
**Service**	
Public	1392 (74.4)
Private	494 (26.6)
**Age (mean, SD)**	43.4 (11.1)
**Sex**	
Female	1427 (77.2)
Male	421 (22.4)
Other	8 (0.4)
**Work experience**	
Years (mean; SD)	18 (11.4)
**Educational background**	
BSc or equivalent title	1280 (69.0)
Postgraduate certificate after BSc	433 (23.6)
Master of Science	127 (6.8)
Postgraduate certificate after MSc	16 (0.8)
**Shift**	
Rotation shifts (day/night)	1170 (63.0)
Day shift only	686 (36.0)
**Intention to leave the ward/service**	
yes	499 (26.9)
**Intention to leave the organization**	
yes	425 (22.9)
**Intention to leave the profession**	
yes	322 (17.3)
**Trust in the leader (mean, SD)**	3.8 (0.7)
Harmony (mean, SD)	3.6 (0.7)
Reliability (mean, SD)	3.8 (0.8)
Concern (mean, SD)	3.9 (0.7)
**Organizational commitment (mean, SD)**	49.4 (21.3)

Legend: SD = standard deviation; BSc = Bachelor of Sciences in Nursing.

**Table 2:** Correlations of study variables (n = 1856).

	Trust Harmony	Trust Reliability	Trust Concern	Overall Trust	Organizational Commitment	Leave Unit	Leave Organization	Leave Profession
Sex	−0.01	−0.03	−0.04	−0.03	−0.01	0.05 *	0.01	0.03
Region	0.04	0.01	−0.01	0.01	0.18 **	−0.01	−0.04	−0.06 **
Setting	−0.07 **	−0.08 **	−0.07 **	−0.08 **	0.08 **	−0.02	0.01	0.04
Shift	0.05 *	0.08 **	0.07 **	0.07 **	0.08 **	−0.07 **	−0.10 **	−0.04
Education	−0.05 *	−0.04	−0.03	−0.04	−0.05 *	0.08 **	0.05 *	0.10 **
Age	0.06 **	0.06 **	0.05 *	0.06 **	0.04	−0.09 **	−0.13 **	0.01
Tenure	0.01	0.01	−0.01	0.01	0.01	−0.05 *	−0.06 *	−0.02
Trust Harmony		0.85 ***	0.79 ***	0.94 ***	0.42 ***	−0.23 ***	−0.28 ***	−0.16 ***
Trust Reliability			0.84 ***	0.96 ***	0.36 ***	−0.27 ***	−0.27 ***	−0.12 ***
Trust Concern				0.93 ***	0.30 ***	−0.25 ***	−.0.23 ***	−0.10 ***
Overall Trust					0.38 ***	−0.27 ***	−0.28 ***	−0.14 ***
Commitment						0.30 ***	0.46 ***	0.34 ***
Leave Unit							0.42 ***	0.28 ***
Leave Organization								0.35 ***
Leave Profession								

Note: The numbers refer to Pearson correlation coefficients (two-tailed). * *p* < 0.05; ** *p* < 0.01; *** *p* < 0.001.

**Table 3:** Path estimates and indirect effects of the mediation model.

	*β*	*p*	SE	95% CI
Direct Effect				
Trust→Commitment	0.42	<0.001	0.02	0.34; 0.45
Trust→Intention to Leave	−0.13	<0.001	0.03	−0.22; −0.08
Commitment→intention to leave	−0.62	<0.001	0.04	−0.78; −0.58
Age→Intention to Leave	−0.14	<0.001	0.01	−0.014; −0.005
Indirect Effect				
Trust→ Intention to Leave	−0.27	<0.001	0.02	−0.33; −0.23
Total Effect				
Trust→Intention to Leave	−0.39	<0.001	0.03	−0.49; −0.35

Note: *p*, level of significance; SE, standard error; CI, confidence interval.

## Data Availability

The datasets presented in this article are not readily available because the data are part of an ongoing study. Requests to access the datasets should be directed to the corresponding authors.

## Public Involvement Statement

There was no public involvement in any aspect of this research.

## Guidelines and Standards Statement

This manuscript was drafted against the STROBE (Strengthening the Reporting of Observational Studies in Epidemiology) guidelines for observational studies.

## References

[1] Alsadaan N., Salameh B., Reshia F.A.A.E., Alruwaili R.F., Alruwaili M., Ali S.A.A., Alruwaili A.N., Hefnawy G.R., Alshammari M.S.S., Alrumayh A.G.R. (2023). Impact of nurse leaders behaviors on nursing staff performance: A systematic review of literature. INQUIRY J. Health Care Organ. Provis. Financ..

[2] Hult M., Terkamo-Moisio A., Kaakinen P., Karki S., Nurmeksela A., Palonen M., Peltonen L.M., Häggman-Laitila A. (2023). Relationships between nursing leadership and organizational, staff and patient outcomes: A systematic review of reviews. Nurs. Open.

[3] Lake E.T., Sanders J., Duan R., Riman K.A., Schoenauer K.M., Chen Y. (2019). A meta-analysis of the associations between the nurse work environment in hospitals and 4 sets of outcomes. Med. Care.

[4] Van der Heijden B., Brown Mahoney C., Xu Y. (2019). Impact of Job Demands and Resources on Nurses’ Burnout and Occupational Turnover Intention Towards an Age-Moderated Mediation Model for the Nursing Profession. Int. J. Environ. Res. Public Health.

[5] Ystaas L.M.K., Nikitara M., Ghobrial S., Latzourakis E., Polychronis G., Constantinou C.S. (2023). The Impact of Transformational Leadership in the Nursing Work Environment and Patients’ Outcomes: A Systematic Review. Nurs. Rep..

[6] Labrague L.J. (2021). Influence of nurse managers’ toxic leadership behaviours on nurse-reported adverse events and quality of care. J. Nurs. Manag..

[7] Karikumpu V., Häggman-Laitila A., Romppanen J., Kangasniemi M., Terkamo-Moisio A. (2024). Trust in the leader and trust in the organization in healthcare: A concept analysis based on a systematic review. J. Nurs. Manag..

[8] Alilyyani B., Wong C.A., Cummings G. (2018). Antecedents, mediators, and outcomes of authentic leadership in healthcare: A systematic review. Int. J. Nurs. Stud..

[9] Kim M., Beehr T.A., Prewett M.S. (2018). Employee responses to empowering leadership: A meta-analysis. J. Leadersh. Organ. Stud..

[10] Kim S., Jeong S.H., Seo M.H. (2022). Nurses’ ethical leadership and related outcome variables: Systematic review and meta-analysis. J. Nurs. Manag..

[11] Putra A.P., Kusnanto K., Yuwono S.R. (2020). Effects of job satisfaction and organizational commitment on nurse retention: A systematic review. Indones. Nurs. J. Educ. Clin. (INJEC).

[12] Xiao Q., Cooke F.L., Chen L. (2022). Nurses’ well-being and implications for human resource management: A systematic literature review. Int. J. Manag. Rev..

[13] Niinihuhta M., Häggman-Laitila A. (2022). A systematic review of the relationships between nurse leaders’ leadership styles and nurses’ work-related well-being. Int. J. Nurs. Pract..

[14] Legood A., van der Werff L., Lee A., Den Hartog D.A. (2021). A Meta-Analysis of the Role of Trust in the Leadership-Performance Relationship. Eur. J. Work Organ. Psychol..

[15] Håvold O.K.S., Håvold J.I., Glavee-Geo R. (2021). Trust in leaders, work satisfaction and work engagement in public hospitals. Int. J. Public Leadersh..

[16] Trybou J., De Pourcq K., Paeshuyse M., Gemmel P. (2014). The importance of social exchange to nurses and nurse assistants: Impact on retention factors. J. Nurs. Manag..

[17] Lim J.Y., Kim G.M., Kim E.J. (2024). Factors associated with turnover intention among hospital nurses: A systematic review and meta-analysis. J. Korean Acad. Psychiatr. Ment. Health Nurs..

[18] Cropanzano R., Anthony E.L., Daniels S.R., Hall A.V. (2017). Social Exchange Theory: A Critical Review with Theoretical Remedies. Acad. Manag. Ann..

[19] Hasnain S.S.S. (2019). Trust-Significance, Definitions and Dimensions: A Literature Search. ABR.

[20] Tzafrir S.S., Dolan S.L. (2004). Trust Me: A Scale for Measuring Manager-Employee Trust. Manag. Res. J. Iberoam. Acad. Manag..

[21] Sasso L., Bagnasco A., Catania G., Zanini M., Aleo G., Watson R., RN4CAST@IT Working Group (2019). Push and pull factors of nurses’ intention to leave. J. Nurs. Manag..

[22] Chen S.-Y., Wu W.-C., Chang C.-S., Lin C.-T., Kung J.-Y., Weng H.-C., Lin Y.-T., Lee S.-I. (2015). Organizational justice, trust, and identification and their effects on organizational commitment in hospital nursing staff. BMC Health Serv. Res..

[23] Nhan N.T., Xuan N.T.T. (2018). Commitment: A concept analysis in nursing field. MedPharmRes.

[24] Hussain M.K., Khayat R.A.M. (2021). The impact of transformational leadership on job satisfaction and organisational commitment among hospital staff: A systematic review. J. Health Manag..

[25] Halter M., Boiko O., Pelone F., Beighton C., Harris R., Gale J., Gourlay S., Drennan V. (2017). The determinants and consequences of adult nursing staff turnover: A systematic review of systematic reviews. BMC Health Serv. Res..

[26] Slater P., Roos M., Eskola S., McCormack B., Hahtela N., Kurjenluoma K., Suominen T. (2021). Challenging and redesigning a new model to explain intention to leave nursing. Scand. J. Caring Sci..

[27] World Health Organization (2016). Global Strategic Directions for Strengthening Nursing and Midwifery 2016–2020. https://iris.who.int/bitstream/handle/10665/275453/9789241510455-eng.pdf?sequence=1.

[28] World Health Organization (2016). Global Strategy on Human Resources for Health: Workforce 2030. https://iris.who.int/bitstream/handle/10665/250368/9789241511131-eng.pdf?sequence=1.

[29] Buchan J., Catton H., Shaffer F. (2022). Sustain and Retain in 2022 and Beyond.

[30] Nei D., Snyder L.A., Litwiller B.J. (2015). Promoting retention of nurses: A meta-analytic examination of causes of nurse turnover. Health Care Manag. Rev..

[31] Hampton D., Welsh D. (2019). Work values of generation Z nurses. J. Nurs. Adm..

[32] von Elm E., Altman D.G., Egger M., Pocock S.J., Gøtzsche P.C., Vandenbroucke J.P., STROBE Initiative (2014). The strengthening the reporting of observational studies in epidemiology (STROBE) statement: Guidelines for reporting observational studies. Int. J. Surg..

[33] Blau P.M. (1964). Exchange and Power in Social Life.

[34] Cropanzano R., Mitchell M.S. (2005). Social Exchange Theory: An Interdisciplinary Review. J. Manag..

[35] Zhou G., Gul R., Tufail M. (2022). Does Servant Leadership Stimulate Work Engagement? The Moderating Role of Trust in the Leader. Front. Psychol..

[36] Lommi M., Notarnicola I., Caruso R., Iacorossi L., Gambalunga F., Sabatino L., Latina R., Rea T., Guillari A., De Maria M. (2023). Psychometric Properties of the Italian Version of the Leader Member Exchange Scale (LMX-7): A Validation Study. Healthcare.

[37] Ivziku D., Caruso R., Lommi M., Conte G., Magon A., Stievano A., Rocco G., Notarnicola I., De Maria M., Gualandi R. (2023). Cultural Adaptation and Psychometric Properties of the Trust Me Scale—Italian Version: A Validation Study. Healthcare.

[38] Lommi M., Caruso R., Conte G., Magon A., Porcelli B., Stievano A., Rocco G., Notarnicola I., Sabatino L., Latina R. (2023). Assessment of the Psychometric Characteristics of the Italian Version of the Nurse Manager Actions Scale. Nurs. Rep..

[39] Cohen J. (1988). Statistical Power Analysis for the Behavioral Sciences.

[40] Williams G.M., Smith A.P. (2016). Using Single-Item Measures to Examine the Relationships between Work, Personality, and Well-Being in the Workplace. Psychology.

[41] Jackson T.A., Meyer J.P., Wang X.H. (2013). Leadership, commitment, and culture: A meta-analysis. J. Leadersh. Organ. Stud..

[42] van Veldhoven M.J.P.M., Prins J., van der Laken P.A., Dijkstra L. (2015). QEEW2.0: 42 Short Scales for Survey Research on Work, Well-Being and Performance.

[43] World Medical Association (2013). World Medical Association Declaration of Helsinki: Ethical Principles for Medical Research Involving Human Subjects. JAMA.

[44] Kline R.B. (2015). Principles and Practice of Structural Equation Modeling.

[45] Brauer K., Ranger J., Ziegler M. (2023). Confirmatory factor analyses in psychological test adaptation and development: A nontechnical discussion of the WLSMV estimator. Psychol. Test Adapt. Dev..

[46] Nunnally J.C. (1978). Psychometric Methods.

[47] Hu L., Bentler P.M. (1999). Cutoff criteria for fit indexes in covariance structure analysis: Conventional criteria versus new alternatives. Struct. Equ. Model..

[48] Byrne B.M. (2006). Structural Equation Modeling with EQS: Basic Concepts, Applications and Programming.

[49] Shi D., Lee T., Maydeu-Olivares A. (2019). Understanding the Model Size Effect on SEM Fit Indices. Educ. Psychol. Meas..

[50] Bobbio A., Manganelli A.M. (2015). Antecedents of hospital nurses’ intention to leave the organization: A cross sectional survey. Int. J. Nurs. Stud..

[51] Gonzalez K.M. (2017). Trust: A Concept Analysis with Watson’s Theoretical Perspective. Nurs. Sci. Q..

[52] Ghahremani H., Lemoine G.J., Hartnell C.A. (2024). The Influence of Servant Leadership on Internal Career Success: An Examination of Psychological Climates and Career Progression Expectations. J. Leadersh. Organ. Stud..

[53] Restivo V., Minutolo G., Battaglini A., Carli A., Capraro M., Gaeta M., Odone A., Trucchi C., Favaretti C., Vitale F. (2022). Leadership Effectiveness in Healthcare Settings: A Systematic Review and Meta-Analysis of Cross-Sectional and Before–After Studies. Int. J. Environ. Res. Public Health.

[54] Cranmer G.A., Rey R.T., Capra J., Browning B., Sollitto M. (2023). Task and Social Determinants of Coaches’ Reports of Leader-Member Exchange. Commun. Sport.

[55] Scandura T.A., Meuser J.D. (2022). Relational Dynamics of Leadership: Problems and Prospects. Annu. Rev. Organ. Psychol. Organ. Behav..

[56] Ferramosca F.M.P., De Maria M., Ivziku D., Raffaele B., Lommi M., Tolentino Diaz M.Y., Montini G., Porcelli B., De Benedictis A., Tartaglini D. (2023). Nurses’ Organization of Work and Its Relation to Workload in Medical Surgical Units: A Cross-Sectional Observational Multi-Center Study. Healthcare.

[57] Ivziku D., de Maria M., Ferramosca F.M.P., Greco A., Tartaglini D., Gualandi R. (2022). What Determines Physical, Mental and Emotional Workloads on Nurses? A Cross-sectional Study. J. Nurs. Manag..

[58] Ivziku D., Ferramosca F.M.P., Filomeno L., Gualandi R., De Maria M., Tartaglini D. (2022). Defining Nursing Workload Predictors: A Pilot Study. J. Nurs. Manag..

[59] Dirks K.T., Ferrin D.L. (2002). Trust in Leadership: Meta-Analytic Findings and Implications for Research and Practice. J. Appl. Psychol..

[60] Östergård K., Kuha S., Kanste O. (2023). Health-care leaders’ and professionals’ experiences and perceptions of compassionate leadership: A mixed-methods systematic review. Leadersh. Health Serv..

[61] Tolksdorf K.H., Tischler U., Heinrichs K. (2022). Correlates of turnover intention among nursing staff in the COVID-19 pandemic: A systematic review. BMC Nurs..

[62] Kang J.Y., Lee M.K., Fairchild E.M., Caubet S.L., Peters D.E., Matti L., Howell T.G. (2023). Do Organizational Values and Leadership Impact Staff Engagement, Wellbeing, and Patient Satisfaction?. J. Healthc. Leadersh..

[63] Bahlman-van Ooijen W., Malfait S., Huisman-de Waal G., Hafsteinsdóttir T.B. (2023). Nurses’ motivations to leave the nursing profession: A qualitative meta-aggregation. J. Adv. Nurs..

[64] International Labour Organization (ILO) (2020). COVID-19 and the World of Work: Impact and Policy Responses.

[65] Stewart D., Catton H., Acorn M., Burton E., Fokeladeh H.S., Parish C., Williamson L. (2022). Nurses: A Voice to Lead. (Invest in Nursing and Respect Rights to Secure Global Health, Issue).

[66] Filomeno L., Forte D., Di Simone E., Di Muzio M., Tartaglini D., Lommi M., Ivziku D. (2024). Systematic Review and Psychometric Properties Analysis of First-, Middle-, and Top-Level Nurse Manager’s Core Competencies Instruments. J. Nurs. Manag..

[67] American Organization for Nursing Leadership (2022). Longitudinal Nursing Leadership Insight Study. https://www.aonl.org/resources/nursing-leadership-covid-19-survey.

[68] Warden D.H., Hughes R.G., Probst J.C., Warden D.N., Adams S.A. (2021). Current turnover intention among nurse managers, directors, and executives. Nurs. Outlook.

[69] Cummings G.G., Lee S., Tate K., Penconek T., Micaroni S.P., Paananen T., Chatterjee G.E. (2021). The essentials of nursing leadership: A systematic review of factors and educational interventions influencing nursing leadership. Int. J. Nurs. Stud..

[70] Frangieh I., Hughes V.D., Mewborn E. (2023). Nurse leaders thriving: A conceptual model and strategies for application. Nurs. Manag..

